# Analysis of Urine Composition in Type II Diabetic Mice after Intervention Therapy Using Holothurian Polypeptides

**DOI:** 10.3389/fchem.2017.00054

**Published:** 2017-07-26

**Authors:** Yanyan Li, Jiajie Xu, Xiurong Su

**Affiliations:** ^1^School of Marine Science, Ningbo University Zhejiang, China; ^2^Department of Food Science, Cornell University Ithaca, NY, United States; ^3^College of Engineering, China Agricultural University Beijing, China

**Keywords:** holothurian polypeptides, function, type II diabetes, urine, express mechanism

## Abstract

Hydrolysates and peptide fractions (PF) obtained from sea cucumber with commercial enzyme were studied on the hyperglycemic and renal protective effects on db/db rats using urine metabolomics. Compared with the control group the polypeptides from the two species could significantly reduce the urine glucose and urea. We also tried to address the compositions of highly expressed urinary proteins using a proteomics approach. They were serum albumins, AMBP proteins, negative trypsin, elastase, and urinary protein, GAPDH, a receptor of urokinase-type plasminogen activator (uPAR), and Ig kappa chain C region. We used the electronic nose to quickly detect changes in the volatile substances in mice urine after holothurian polypeptides (HPP) fed, and the results show it can identify the difference between treatment groups with the control group without overlapping. The protein express mechanism of HPP treating diabetes was discussed, and we suggested these two peptides with the hypoglycemic and renal protective activity might be utilized as nutraceuticals.

## Introduction

Diabetes mellitus (DM), a major epidemic of this century (Shaw et al., 2010; Forbes and Cooper, 2013), is a metabolic syndrome characterized as high blood sugar, which is caused by disorder of insulin secretion or functioning. A persistent high blood sugar and long-term metabolic syndrome may cause chronic damage and functional disorder of multiple organs (Brownlee, 2001; Tabak et al., 2012). Diabetic nephropathy is one of the important complications of diabetes, with glomerular damage being the dominant feature in early stage (Rosolowsky et al., 2011; Stec et al., 2015). Initial urine is the first produced by glomerular filtration of blood after processing in the kidney, and then urine is produced by reabsorption, excretion, and secretion by renal tubules and collecting tubules. Urinary proteins, comprising a variety of biomarkers, are the mixture of plasma proteins and kidney proteins, of which the former contributes 30% and the latter the remaining 70% (Wearn and Richards, 1924). The changes of the composition and amount of urinary proteins are indirect indicators of the nature and degree of kidney diseases (Bruce et al., 2009; Klein et al., 2016; Modena et al., 2016; Onuh et al., 2016). Therefore, by profiling the urinary proteins and the small-molecule substances, some special components may be observed. They can be used as specific molecular markers in the diagnosis, monitoring, mechanism research, and new drug development for diabetes and kidney diseases (Good et al., 2010; Papadopoulos et al., 2016).

Previous studies mostly focus on large proteins and polypeptide molecules in the urine while paying no attention to small-molecule compounds (Fu et al., 2016; Kraus et al., 2016). Some volatile compounds produce distinctive odor after entering the urine. So they can be used as indicators of early diagnosis and treatment of diabetes.

*Apostichopus japonicus* (also named as *Stichopus japonicus*, hereafter referred to as *A. japonicus*) and *Acaudina molpadioides* (hereafter referred to as *A. molpadioides*), both common named Sea cucumber in China, belong to Class Holothuroidea of Phylum Echinodermata (Vieira and Mourao, 1988). Previous experimental results indicated that the two holothurians contain low fat and high protein levels (Table S1). In addition to having a high nutritious value, sea cucumbers have long been recognized in the folk medicine system of Asian countries (Bordbar et al., 2011). The polypeptides formed via acid hydrolysis can effectively scavenge free radicals in animals, reduce triglyceride and cholesterol levels, increase essential trace element contents, accelerate hemoglobin and erythrocyte production, and improve circulation (Vieira et al., 1991; Wu et al., 2012). These polypeptides also induce mild and persistent multi-target and multi-hierarchy therapeutic effects and regulatory functions without causing side effects. So there were several studies of Sea cucumbers on the maintain glycemic control or therapeutic effect on DM. The results showed that the Sea cucumbers or the polypeptides had the ameliorative effect on insulin resistance and kidney damage, could remarkably reduce blood sugar. Yet, the precise mechanisms underlying such beneficial effect remained unclear. Our present study thus aimed to address the effect of holothurian polypeptides (HPP) on excretory level and compositions of urinary proteins using a proteomics approach.

In light of this, a 6-month clinical intervention was administered in leptin receptor-deficient (db/db) mice using polypeptides extracted from holothurians. The proteins and small-molecule substances in mouse urine were profiled by two-dimensional electrophoresis, electronic nose, HS-SPME-GC-MS, and nuclear magnetic resonance (NMR). The molecular mechanism of HPP treating diabetes was discussed, and a foundation was laid for clinical diagnosis of diabetes.

## Materials and methods

### Ethics statement

This study was approved by the Ethical Committee of China Pharmaceutical University, Nanjing University, the Laboratory Animal Management Committee of Jiangsu Province (Approval No.: 2110748), and the Ethical Committee of Ningbo University, Zhejiang Medical Animal Care Committee at Hangzhou (Approval No.: 2008-0110). The animal experiments were conducted in compliance with the standard ethical guidelines for the control of these ethics committees.

### Materials

C57BL/6J mice (20 ± 1.0 g, 4–6 weeks) and leptin receptor-deficient (db/db) mice (30 ± 2.0 g, 4–6 weeks) were purchased from Shanghai Bolaite Co., Ltd. [Licensed ID, SCXK(HU)2007-0005, invoice number, 2007000528007]. IPG dry strip (linear 3–10 L, 7 cm), IPG buffer (pH 3-10), low-melting-point agarose for sealing, urea, dithiothreitol (DTT), CHAPS, sodium dodecyl sulfate (SDS), Tris, iodoacetamide, and mineral oil were purchased from Bio-Rad Company. Acrylamide, ammonium persulfate (APS), TEMED, bisacrylamide, glycerol, Coomassie brilliant blue R-250, and glycine were purchased from Ameresco Company. Other reagents were purchased from Sangon Biotech (Shanghai) Co., Ltd. The polypeptides from *A. molpadioides* (nitrogen content 93.73 mg g^−^^1^) and *A. japonicus* (nitrogen content 86.46 mg g^−^^1^) (the component of the holothurians shown in Table S1, Figure S1) were prepared by our laboratory.

### Preparation of the holothurian polypeptides (HPP)

The holothurian was rinsed with distilled water and homogenized, and then 600 mL of deionized (DI) water was added to 100 g of the holothurian. The homogenate was digested with 1.8% (w/v) Protamex (1.0 × 10^5^ U g^−^^1^) at 55°C for 136 min.

After the reaction, the solutions were placed in a boiling water bath for 10 min to inactivate the enzyme. The hydrolysates were clarified by centrifugation at 12,000 g for 10 min at 4°C to remove the unhydrolyzed residue. Finally, the HPP hydrolysates were obtained after filtering the supernatant, followed by storage at −25°C until use.

### Amino acid analysis of HPP

The amino acid profile of HPP was determined according to the method of Ding et al. (2011). The amino acid composition was determined by high-performance liquid chromatography (Agilent, 1200, USA). The total amino acids (except for tryptophan) were determined after hydrolysis at 130°C for 40 min with 6 M HCl by the microwave digestion system (Mars, CEM, USA). Alkaline hydrolysis was also done for the determination of tryptophan level. The amino acid standards (Sigma, USA) were using the same conditions as the samples.

### Grouping and rearing

The experimental groups were as follows: blank control group (C) consisting of 10 C57BL/6J male mice. The db/db mice were randomly assigned to four groups of 10 animals each, model group (M), positive drug group (Me), *A. molpadioides*'s polypeptide group (A), and *A. japonicus*'s polypeptide group (S). For Group C, deionized water of equal amount as the experimental group was perfused. For Group Me, methyl oleanolate was perfused at the rate of 3 mg kg^−^^1^ d^−^^1^. For the two experimental groups, the polypeptides from *A. molpadioides* and *A. japonicus* were separately administered at the dose with of 50 mg kg^−^^1^ d^−^^1^. The mice of all groups were allowed free access to food and water (Table S3). The room temperature was maintained at about 25°C, and 12 h/12 h day and night alternation were practiced (Xu et al., 2017). The mice were administered with drug for 6 months (The difference of urinary protein wasn't significant until 3.5 months between Group C and Group M. We treated the experimental group for another 2.5 months to guarantee the therapeutic effect). Urine samples were collected from the metabolic cages after fasting for 12 h. The feces residue was filtrated with filter paper, sub packaged, and freezed at −80°C.

### Extraction of urinary proteins

The urine sample was centrifuged (20 min, 6,000 × g, 4°C). Then the supernatant was filtrated using 0.45 μm membrane (Millipore, German). The filtrate was dissolved in acetone and precipitated overnight, and solution was centrifuged (20 min, 6,000 × g, 4°C). The supernatant was discarded, and the precipitate was dissolved in Tris-saturated phenol. After centrifuging again (20 min, 12,000 × g, 4°C), the upper layer containing the phenol was added with ammonium acetate/methanol solution with five times volume of itself to precipitate the proteins at 4°C overnight. The precipitate was centrifuged (20 min, 12,000 × g, 4°C). The supernatant was discarded, and 1 mL of methanol solution was added. The proteins were repeatedly blown and washed. Following centrifugation at 12,000 g for 20 min, the supernatant was discarded, and 1 mL of methanol solution was added into the precipitate to repeatedly blow and wash the proteins. The product was then subject to centrifugation at 12,000 g for 20 min, and the supernatant was discarded. The precipitate was stored at −80°C.

### Two-dimensional electrophoresis

The hydration loading buffer freezed at −20°C was taken out, and 9.8 mg DTT and 5 μL Bio-Lyte were added into each milliliter. The protein sample of 300 μg was fully mixed with 200 μL of hydration solution. The IPG strips (24 cm, pH 4–7) freezed at −20°C were taken out from the fridge and placed at room temperature for 10 min. The sample was added along the groove of focusing plate. Mineral oil of about 1 mL was applied on each strip, and isoelectric focusing was conducted. Subsequently, the strips were taken out, placed into the balancing liquid for 15 min under shaking. Then the balanced strips were transferred to the upper end of 12% gel. The initial voltage was 90 V and was later raised to 200 V when the sample swam out of the stacking gel. Vertical SDS-PAGE electrophoresis as the second dimension was run until bromophenol blue reached the bottom of the gel.

### Mass spectrometry and data analysis

After electrophoresis, the strips were immobilized, stained, destained, and added with Coomassie brilliant blue R-250 on a shaker at room temperature for 90 min. Scanning was performed using BioRad GS-800 calibrated densitometer, and PDQuest software was used for data analysis. The protein spots with good repeatability, clear expression, and definite boundary were automatically selected. Spot location data was stored in a relational database and retrieved by a proprietary cutter. The visible stained gel spots were systematically cut out and collected into bar-coded 96-well-microtiter plates for further processing. The differential protein spots were cut off the gel and analyzed by Bruker Dalton Autoflex speed™ MALDI-TOF-TOF mass spectrometer, which was equipped with delayed ion extraction, pulsed nitrogen lasers (10 Hz Biflex, 20 Hz Autoflex), dual microchannel plates and 2 GHz transient digitizers. All mass spectra represented signal averaging of 120 laser shots. The performance of the mass spectrometers had sufficient mass resolution to produce isotopic multiplets for each ion species below m/z 3,000. Spectra were internally calibrated using two spiked peptides (angiotensin II and ACTH18–39) and database-searched with a mass tolerance of 50 ppm. For protein identification, the NCBI database was searched by BioTools (Bruker Dalton) software to look for the matched proteins, the functions of which were also inquired.

### Odor detection of urine using electronic nose

Mouse urine of 200 μl was sealed in 15 mL vial with a threaded neck, insured the vials' tightness, and heated for 30 min in 60°C water bath, and stood at room temperature for 15 min for balancing. Five replicates were set up for each sample. Detection was carried out using electronic nose (PEN3, Airsense, Germany). The detection lasted for 150s–300s, and the sensor was cleaned automatically (Sun et al., 2013).

Several indicators with great correlation in the results detected by electronic nose were converted into synthetic indicator. In principal component analysis (PCA), when the contribution rate of the two principal components exceeded 85%, it was considered that the two principal components represented the features of the original experimental data; otherwise, it was considered that there were interfering components. The data collected in 199s–200s were selected for PCA, Least absolute deviation (LAD) analysis based on the built-in software coming with the equipment.

### Analysis of volatile compounds in the urine by HS-SPME-GC-MS

#### Extraction

The extractor (65 μm PDMS, SUPELCO, USA) was placed near the injection port of gas chromatograph for aging at 250°C for 30 min. Then the extractor was inserted into the sample flask in 60°C water bath for adsorption for 30 min. Desorption was performed at 220°C for 5 min by transferring the extractor to the injection port of gas chromatograph-mass spectrometer (7890/M7-80EI, Agilent, USA, Beijing Persee General Instrument Co., Ltd.). The gas chromatograph-mass spectrometer was turned on to collect data.

#### Chromatography

DB-5 capillary column (30 m × 0.25 mm × 2.5 μm), carrier gas He with flow rate of 1 mL min^−1^, sample injection in splitless mode for 1 min at the constant rate of 1 mL min^−1^; the temperature of the injection port and the cable was both 220°C. Programmed temperature rising: initial column temperature 50°C, raised to 200°C at 5°C min^−1^ and maintained for 5 min; then raised to 250°C at 10°C min^−1^ and maintained for 2 min.

#### Mass spectrometry

The electron impact (EI) ion source was used, with ionization voltage of 70 eV, ion source temperature of 230°C and scanning range of 45–400 μm.

GC-MS detection results were searched with computer. Moreover, qualitative analysis was performed by mutual matching between NIST and WILEY mass spectral libraries. The compounds in the mass spectral libraries with identity lower than 80 (the maximum being 100) were marked as unidentified. The relative percentage content of each component was used to calculate relative content by peak area normalization method.

### NMR analysis of small-molecule metabolites in mouse urine

The mouse urine was added with methanol and then centrifuged (10 min, 12,000 × g, 4°C). The supernatant was collected, and the methanol was removed using nitrogen blowing sample concentrator. Then the product was freezed, dried, and pulverized for detection by 1H NMR.

Phosphate buffer (0.1 M, 10% D2O, pH 7.4, 1.5 mM TSP) 600 μL was added into the dry powder of mouse urine extract, and vortex oscillation was performed for 30 s. Then the sample was completely transferred onto the ultrafiltration membrane for centrifugation for 15 min at 4°C at 13,000 g. This procedure was repeated twice, and 450 μL of transparent filtrate was added into 50 μL of Anachro certified DSS standard solution (ACDSS). The tube was vortex oscillated for 10 s at 13,000 g, followed by centrifugation for 2 min at 4°C. Then 480 μL of supernatant was placed into the NMR tube and detected using Bruker Avance III 600 MHz NMR spectrometer (Bruker Biospin, Rheinstetten, Germany). The experimental temperature was 298 K, and proton resonance frequency was 600.13 MHz. Pulse sequence Noesygppr1D was used to collect 1H NMR spectra. Water peak was suppressed, and the spectral width was set as 20 ppm. There were 32 K sampling points and free induction decay (FID) signals were accumulated for 64 times.

The FID signals of 1H NMR were imported into Chenomx NMR suite (version 7.6, Chenomx, Edmonton, Canada). Fourier transform was performed automatically with phase adjustment and baseline calibration. DSS-d6 peak (0.0 ppm) was considered as the standard for the chemical shift of all spectra, on which inversion and convolution was performed and the peak shape (chemical shape indicator, CSI) was adjusted. The information of signals in 1H NMR spectra (chemical shift, peak shape, peak width at half height and coupling and splitting) was read. The concentration at and the area of DSS-d6 peak were considered as the standard. Signal analysis was carried out combining with Chenomx database, so as to obtain the type and concentration of each metabolite. The data were subject to logarithmic conversion and median normalization. Moreover, PCA and partial least squares discrimination analysis (PLS-DA) were carried out.

## Results

### Amino acid composition of HPP

The amino acid composition of HPP are abundant and comprehensive, and the contents of aspartic acid, arginine, glutamic acid, lysine, cysteine, and serine were high (Table 1). Some amino acids, especially the branched chain amino acids, can improve the exercise capability and markedly retard the catabolism of protein in the muscle during exercise (You et al., 2011). Aspartic acid was considered to be helpful in the oxidative determination and could lower the blood ammonia concentration, therefore delaying the occurrence of fatigue (Ding et al., 2011). Glutamic acid was found to have a very positive effect on the nervous system and would also be helpful during exercise (Rothman and Olney, 1986).

**Table 1:** The amino acid composition of HPP.

**A.A**.	***A. molpadioides***		***A. japonicus***	
	**Contents (g kg^−1^)**	**Percentage (%)**	**Contents (g kg^−1^)**	**Percentage (%)**
Asp	2.1 ± 0.12	16.22	2.4 ± 0.11	19.15
Glu	1.9 ± 0.11	14.67	2.2 ± 0.13	17.55
Ser	0.6 ± 0.04	4.63	0.45 ± 0.09	3.59
His	0.43 ± 0.03	3.32	0.16 ± 0.07	1.27
Gly	1.9 ± 0.07	14.67	1.4 ± 0.05	11.17
Thr^*^	0.61 ± 0.04	4.71	0.73 ± 0.03	5.82
Ala	1.8 ± 0.09	13.90	1.1 ± 0.07	8.77
Arg	0.82 ± 0.04	6.33	0.94 ± 0.07	7.50
Tyr	0.35 ± 0.03	2.70	0.35 ± 0.02	2.79
Cys	1.2 ± 0.09	9.26	0.7 ± 0.01	5.58
Val^*^	0.11 ± 0.02	0.84	0.71 ± 0.02	5.66
Met^*^	0.25 ± 0.03	1.93	0.2 ± 0.03	1.59
Phe^*^	0.32 ± 0.03	2.47	0.08 ± 0.02	0.63
Ile^*^	0.23 ± 0.01	1.77	0.29 ± 0.07	2.31
Leu^*^	0.091 ± 0.01	0.70	0.54 ± 0.06	4.30
Lys^*^	0.074 ± 0.03	0.57	0.18 ± 0.02	1.43
Pro	0.16 ± 0.04	1.23	0.1 ± 0.05	0.79

* *Essential amino acid. A.A., Amino acid*.

### Differentially expressed proteins in mouse urine

**Figure 1:**
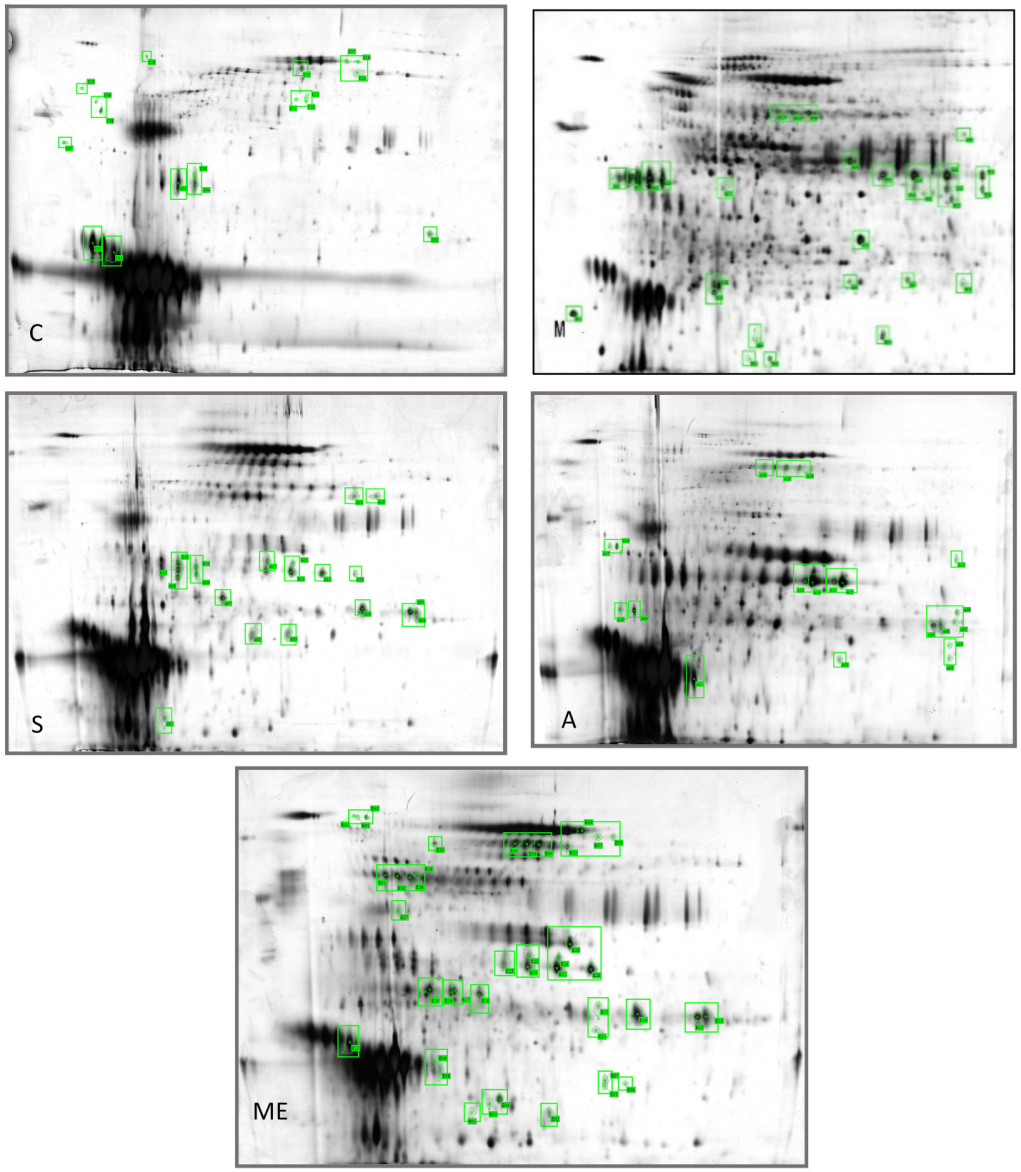
Two-dimensional electrophoregram. C, Control group; M, model group; Me, positive drug group; A, *A. molpadioides's* polypeptide group; S, *A. japonicus's* polypeptide group.

Profiling was carried out using 2D-E technique on the differential proteins in the urine of type II diabetic mice (db/db) after the interference by the polypeptides of *A. molpadioides* and *A. japonicus*. As analyzed data (Figure 1), there were 685 ± 9 protein spots in Group C, and the average intra-group matching rate was 65%. The number of protein spots in Group M was 1,322 ± 50, and the average intra-group matching rate was 81.7%. The number of protein spots in Group Me was 789 ± 36, and the average intra-group matching rate was 74.3%. The number of protein spots in Group A was 865 ± 43, and the average intra-group matching rate was 65.3%. The number of protein spots in Group S was 807 ± 131, and the average intra-group matching rate was 62.3%.

The analysis of the intersection of differential proteins in Group C, experimental groups and model group showed that mouse urine contained 20 proteins. All five groups had serum albumin. Group C, S, and M had major urinary proteins 8 and 11; Group Me, S, and M had major urinary protein 2; Group A and M had anionic trypsin-2; only Group C had the fragment of major urinary protein 3; only Group Me had Ela3 and protein AMBP; Group S had Napsin-A. In Group M, 13 highly expressed differential proteins were identified. Among them, glyceraldehyde-3-phosphoglyceraldehyde dehydrogenase (GAPDH) (accounting for 50%) was the predominant, followed by urokinase-type plasminogen activator (uPAR), and immunoglobin K chain. The isoelectric point of the proteins was basic (pH 6.61–8.98). Three identified proteins were the marker proteins of kidney diseases. In Group A, four differential proteins were serum albumin, and there were one urinary protein and one trypsin carrying one negative charge. The isoelectric point of the proteins was acidic (pH 4.40–5.75). There were two serum albumins and one urinary protein in Group S, and the isoelectric point of the proteins was acidic (pH 4.85–5.75). In Group Me, there were 12 serum albumins, 1 microglobulin, 1 elastase precursor, and 2 urinary proteins. The isoelectric point of the proteins was acidic (pH 5.75–5.85). The standard protein profiles are established with the normal urine which can be referred to when monitoring the changes of urinary proteins in disease conditions (Weissinger et al., 2014). In the experimental groups, treated with sea cucumber, the urinary proteins with high abundance are serum albumins, AMBP proteins, negative trypsin, elastase, and urinary protein, GAPDH, receptor of uPAR, and Ig kappa chain C region. The information and the location of these proteins were provided in Table S4.

### Volatile compounds detect by electronic nose

**Figure 2:**
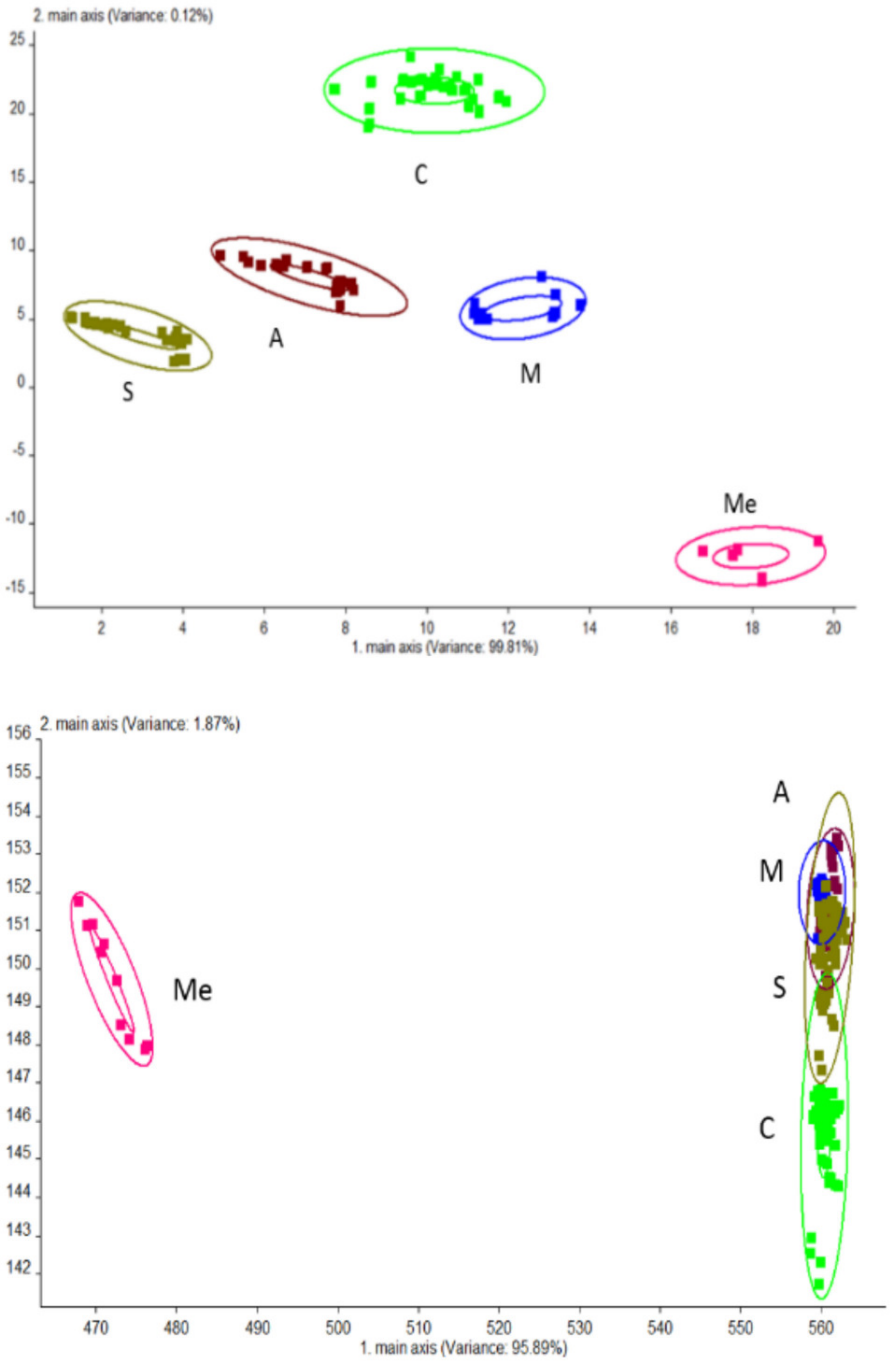
Odor analysis of urine samples of db/db mice using electronic nose. C, Control group; M, model group; Me, positive drug group; A, *A. molpadioides's* polypeptide group; S, *A. japonicus's* polypeptide group. **(Top)** PCA; **(Bottom)** LDA.

PCA analysis was performed for urine samples collected from different groups using electronic nose, and the results are shown in Figure 2. Using the established model, the data of Group C, M, Me, A, and S were located in their respective regions without overlapping. This indicated significant differences in odor among different groups and the presence of a large amount of volatile compounds. The contribution rates of PC1 and PC2 were 99.81 and 0.12%, respectively. It was indicated that the extracted information could reflect the majority of the information contained in the original data. PCA is suitable for the differentiation of urine collected from different experimental groups. LAD regression showed that Group ME was distant away from other groups. This indicated that Group Me greatly differed from other groups in terms of odorous volatile compounds.

### Volatile compounds detect by HS-SPME-GC-MS

**Figure 3:**
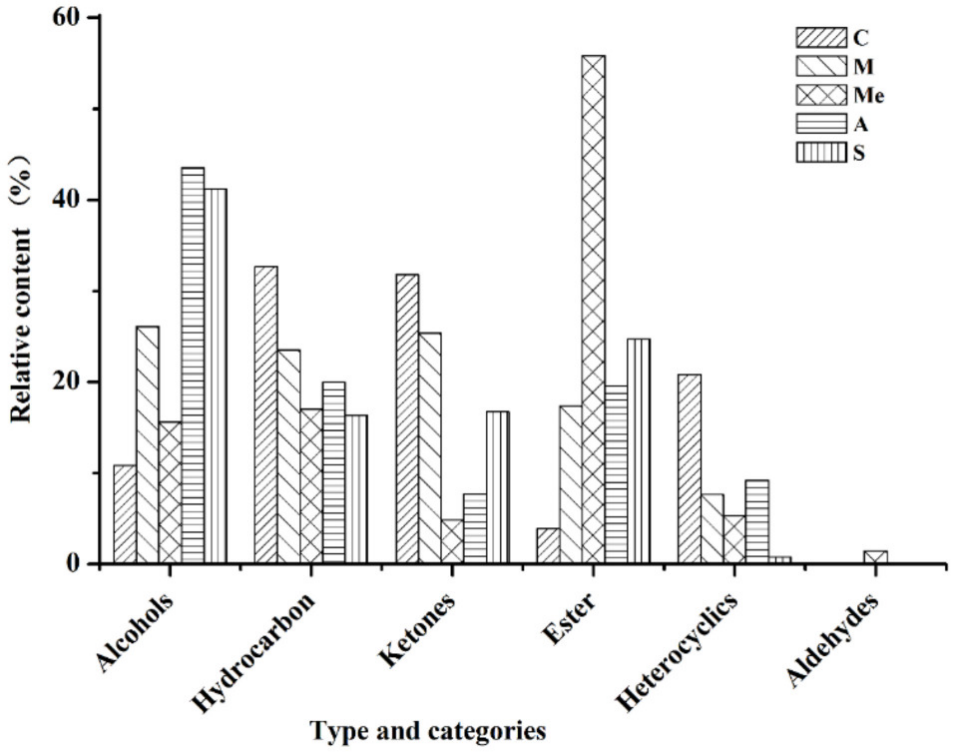
Volatile compounds in the urine of db/db mice. C, Control group; M, model group; Me, positive drug group; A, *A. molpadioides's* polypeptide group; S, *A. japonicus's* polypeptide group.

The volatile compounds of five groups were analyzed by GC-MS eluting peak and searched in NIST and WILEY libraries (Figure 3). In the blank control group, 28 compounds were detected. Among them, there were 4 alcohols (10.81%), 6 hydrocarbons (32.66%), 11 ketones (31.77%), 2 esters (3.88%), and 5 other compounds (20.84%). In Group M, 26 compounds were detected, including 5 alcohols (26.04%), 6 hydrocarbons (23.51%), 7 ketones (25.36%), 5 esters (17.37%), and 3 other compounds (7.65%). In Group Me, 27 compounds were detected. They were 4 alcohols (15.57%), 8 hydrocarbons (16.99%), 4 ketones (4.83%), 1 aldehyde (1.46%), 4 esters (55.81%), and 5 other compounds (5.31%). In Group A, 20 compounds were detected. They were 4 alcohols (43.53%), 5 hydrocarbons (19.98%), 3 ketones (7.73%), 4 esters (19.57%), and 4 other compounds (9.18%). In Group S, 18 compounds were detected. They were 3 alcohols (41.18%), 7 hydrocarbons (16.33%), 5 ketones (16.72%), 2 esters (24.71%), and 1 other compound (0.77%). It can be seen from Figure 3 that the contents of hydrocarbons and ketones were the highest in blank control group; the contents of alcohols, hydrocarbons, and ketones were the highest in Group M; the content of esters was the highest and that of ketones was the lowest in Group Me with trace amount of aldehydes. The content of alcohols was the highest in Group A, followed by hydrocarbons and esters.

The results of solid-phase microextraction (SPME) and GC-MS showed (Table S2) that all urine samples had the odor of lilac (α-Terpineol). The urine samples in the blank control group had the odor of pear (2-heptanone), green grass (3-hepten-2-one), fruit (6-methyl-5-hepten-2-one), and hawthorn (acetophenone). The urine samples in Group M had the mixed odor of orange (2-undecanone), and pungent odor (acetone). The urine samples in Group Me had the odor of almond (2-nitro-benzaldehyde). The urine samples in Group A had a strong odor of guaiacol with a mild sweet odor (4-ethyl-phenol). The urine samples in Group A had a heavy odor of sandalwood (cedrol).

### Small-molecule metabolites

**Figure 4:**
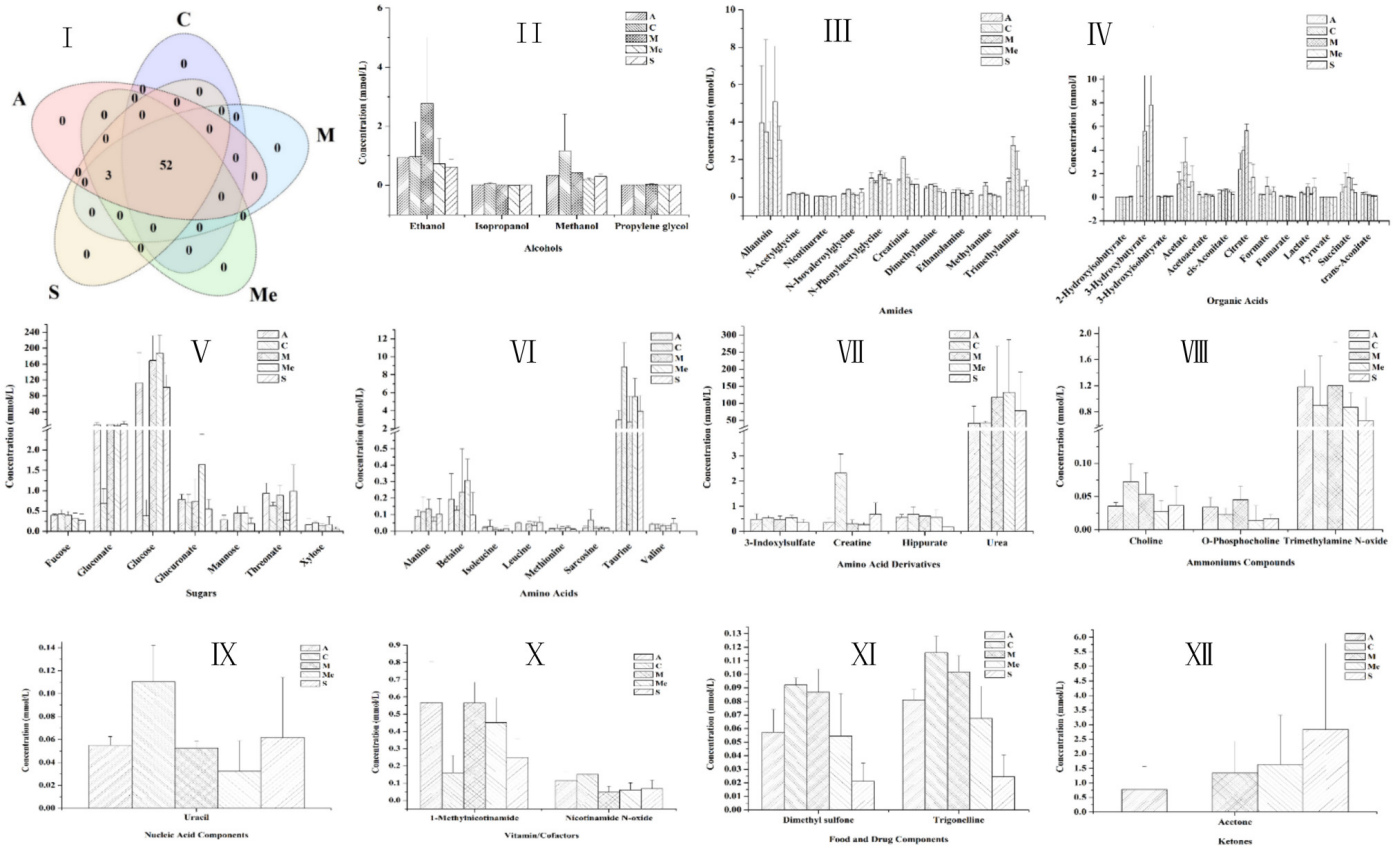
Contents of metabolites in the urine of db/db mice. C, Control group; M, model group; Me, positive drug group; A, *A. molpadioides's* polypeptide group; S, *A. japonicus's* polypeptide group. I, Analysis of intersection of metabolites in each group; II, Alcohols; III, Amines. Additives; IV Organic acids; V, Sugar; VI, Amino acids; VII, Amino acid derivatives; VIII, Ammoniums; IX, Nucleic acids; X, Vitamins; XI, Additives; XII, Ketones.

The 1H NMR spectra of the metabolites of urine samples in each group were analyzed, and 56 metabolites were detected (Figure 4). They were divided into 10 categories: alcohols, amides, amino acids, ammoniums compounds, chemical shape indicator, food, and drug components, ketones, nucleic acid, organic acids, sugars and vitamin cofactors. The five groups all had 52 metabolites, while acetoacetate, acetone, and leucine were unique to the groups of diabetic mice. For Group M, the contents of glucose, gluconate, urea, 3-hydroxybutyrate, 3-hydroxyisobutyrate, 2-hydroxy-isobutyrate, citrate, acetate, succinate, cis-aconitate, formate, lactate, fumarate, ethanol, propylene glycol, alanine, N-phenylacetylglycine, 1-methylnicotinamide, O-phosphocholine, trimethylamine N-oxide were obviously higher. The HPP could obviously lower the contents of urine sugar and urea with a better effect compared with the Group Me. The effect of polypeptide from *A. japonicus* was better than that from *A. molpadioides*, with almost the same content as that in Group C.

## Discussion

The receptor of urokinase-type plasminogen activator (uPAR) is also known as CD87 consisting of 7 exons and 6 introns. It has no transmembrane and intracellular region, belonging to single-strand glycosylated protein. In the rat model of glomerulonephritis, besides the deposition of a large amount of extracellular matrix in the glomeruli, there was also an obvious upregulation of uPAR in the endothelial cells, mesangial cells, and podocytes. uPAR participates in the damage and shedding of podocytes (Vasir et al., 2000). uPAR is not only expressed on the membrane of a variety of immune cells, but also in the intrinsic tissue cells. Wei et al. found that the expression of uPAR was upregulated in the glomeruli in diabetic nephropathy. Moreover, uPAR was also highly expressed on the membrane of podocytes in the mouse model of kidney diseases induced by lipopolysaccharide and puromycin (Wei et al., 2011). It was found in the diabetic mouse model treated by HPP that uPAR in the urine of model group was highly expressed, which was associated with the shedding of podocytes caused by diabetes.

Ig kappa chain C region is the Ig light chain constant region, localized on Ig light chain. It is a spherical structure composed of 110 amino acids, presenting such biological characteristics as immunogenicity, ability to penetrate the placenta and to bind to the complement. Ig kappa chain C region is the hydrolyzed fragment of Ig. When existing alone, it performs no biological functions. If detected in large quantities in the urine, it is indicated that protein filtration was enhanced. Many studies have proved that the damage of kidney functions can lead to enhanced filtration of immunoglobulin in the urine. Fragment of Ig kappa chain C region was detected in the urine sample of the model group. This indicated that the glomerular filtration was impaired in the model group. However, similar phenomenon was not detected in other experimental groups (Khamlichi et al., 1992).

Elastase is a single polypeptide consisting of 240 amino acids. It can specifically decompose collagen and elastin and regulate the collagen and elastin in human arterial wall and connective tissues. Diabetic nephropathy is characterized by the thickening of glomerular basement membrane in terms of pathological changes. One of the key chemical components of glomerular basement membrane is collagen. Elastase can decompose the N-terminal bridge into benzene hexacarboxylic acid and collagen, thus inhibiting the formation of fibers from collagen. It also has an inhibitory effect on the thickening of glomerular basement membrane in diabetic mice. Besides, elastase can improve lipid metabolism. In the treatments of albuminuria, elastase could reduce albuminuria, and improve the kidney diseases. Elastase has a certain preventive effect in diabetic nephropathy. The working mechanism can be described as hydrolysis of elastin to regulate the metabolism of elastin in the artery and the connective tissues. In the present study, the elastase gene was overly expressed in the urine of positive drug group. The reason is that positive drug recovered the secretory function of the pancreas.

Trypsin is divided into cationic and anionic trypsin (about 2:1). Once activated, the cationic trypsin gives rise to two active peptides, which are Ala-Pro-Phe(Asp) 4-Lys and (AsP) 4-lys. In contrast, the activated anionic trypsin only gives rise to an active hepapeptide. The two types of trypsin had no immunologic cross-reactivity. Adrian found that healthy people would have a significant increase of trypsin in the small intestines after eating, but there was no obvious rise of cationic trypsin concentration in the blood (Adrian et al., 1979). The reason is that once entering the small intestines, trypsin can be quickly activated, then bind to the trypsin inhibitor in the blood, and is finally removed by the reticuloendothelial system. However, the exogenous trypsin is directly released into the blood by the pancreas. For insulin-dependent diabetes, the activity of serum trypsin is associated with symptom alleviation. In the urine of mice treated by the polypeptide from *A. molpadioides*, the activity of anionic trypsin was obviously elevated, which may be associated with the restored activity of the pancreas. *A. molpadioides* contains a large amount of collagen, exhibiting anti-oxidative and anti-fatigue functions.

The human metabolism database contains rich information that demonstrates the highly complex chemicals in human urine, some of which are not to be found in the blood (Bingol and BrüSchweiler, 2015). The metabolites of urine usually include the pollutants from food, drink, drugs and environment, metabolic products of human body, and the by-products of bacteria. Compared with other body fluids such as saliva or cerebrospinal fluid, the urine contains compounds over 5–10 times volume, which indicates the diversity of chemicals in the urine (Beckonert et al., 2007). It was previously held that the chemicals in the urine only constitute a subset of compounds in the blood. In fact, the presence of such a large quantity of compounds unique to urine seems to imply the special role of kidney in the filtration of blood. The compounds in the urine are associated with diet, environment, and physiological status. We found that the urine odor was greatly different in differently fed mice by SPME/GC/MS. These urine samples could be quickly differentiated by the electronic nose, and the results had no overlapping. Although the electronic nose cannot determine the specific type of substance, it can memorize the odor information and then establish the template by sensing and recognizing the odor. In this way, different odors can be identified. This function of electronic nose is of great significance for disease diagnosis and efficacy evaluation of drugs. SPME/GC/MS results indicated that the urine samples in the blank control group had the odor of pear (2-heptanone), green grass (3-hepten-2-one), fruit (6-methyl-5-hepten-2-one), and hawthorn (acetophenone). The pungent odor came from acetone in the urine samples in the model group, while the almond-like odor came from 2-nitro-benzaldehyde in the urine samples in the positive drug group. The urine samples in Group A had the strong odor of guaiacol with a mild sweet odor (4-ethyl-phenol). Cedrol with the odor of sandalwood in Group S was the special fingerprint. The content of cedrol can be used to determine the occurrence, progress and treatment condition of diabetes.

As indicated by previous researches, type II diabetes is usually associated with a rise of content of the following substances: n-butryic acid, 1-hydroxy-2-butryic acid, B-hydroxybutyric acid, citrate, acetate, trimethylamine, dimethylamine, trimethylamine N-oxide, acetoacetate, N-N-dimethylglycine, alanine, ornithine, phenylalanine, taurine, betaine, hippurate, and N-methyl-nicotinamide. The contents of the following compounds decline: sarcosine, creatinine, glutamate, fumaric acid (fumarate), malic acid, 2-oxoglutaric acid, succinic acid, tyrosine, histidine, isoleucine, leucine, tryptophan, hydantoin, N-methylnicotinate, and uridine N-methyl-2-pyridine-5-hydroxyamide (Brownlee et al., 1980). Gluconeogenesis in liver will lead to the rise of amino acids in the urine in diabetic patients. In the meantime, the changes of glomerular filtration rate and stress reaction will cause the changes of taurine level. The decomposition of amino acids and proteins will in turn induce the changes of histidine level. Organic acids containing 5 or 6 carbons are the intermediates of tricarboxylic acid cycle. Their changes will hinder the energy supply in diabetic patients, or even lead to the production of ketones or the intermediates of ketones. The increase of citrate is also related to tubular secretion. The unbalance between trimethylamine and dimethylamine means that the formate metabolism is disturbed. The rise of betaine and the decline of hydantoin indicate that the diabetic cases may be complicated by kidney injury. This may be caused by the high permeability of glucose and disorder of papillary function in nephropathy. Glomerular filtration rate has an influence on the concentrations of hydantoin and creatinine. These changes conform to the pathological condition of diabetic cases. Insulin insufficiency will weaken the activity of some enzymes and accordingly the tricarboxylic acid cycle. As a result, the disorder of protein metabolism will occur. The protein synthesis in the muscles and liver will decline, while decomposition will be enhanced. As a result, the concentration of amino acids in the plasma and urine will rise. HPP obviously lowered the contents of 2-hydroxyisobutyrate, 3-hydroxy-butyrate, and 3-hydroxyisobutyrate. These compounds are the most important constituents of ketones with the highest acidity. They are usually chosen as the reliable indicators of diabetic ketoacidosis with potential lethality. They are very effective for the early treatment of diabetic ketoacidosis, as claimed by previous studies (Lebovitz, 1995; Morris et al., 1997; Elliott et al., 2014). An obvious rise of nicotinamide N-oxide may serve as a new biomarker of the progression of type II diabetes. In our experiment, the HPP greatly increased the content of creatinine in mouse urine, especially after the administration of polypeptide from *A. japonicus*. Moreover, the contents of urea and glucose were also lowered considerably. The content of urea nitrogen is determined by the balance between urea production and excretion. As the main end product of human protein metabolism, urea nitrogen is one of the indicators of kidney function. Its upregulation may indicate the occurrence of diabetic nephropathy (McCullough et al., 1997; Elliott et al., 2014).

## Author contributions

XS proposed the study, and provided guidance to all co-authors. YL and JX: designed and conducted the experiment; collected samples. YL performed all methods, including corresponding data analysis and figure preparation. JX prepared other figures. XS, YL wrote the manuscript, with revisions from all the other authors.

### Conflict of interest statement

The authors declare that the research was conducted in the absence of any commercial or financial relationships that could be construed as a potential conflict of interest.
