# Supplementary material for: Analysis of Urine Composition in Type II Diabetic Mice after Intervention Therapy Using Holothurian Polypeptides
Source: Front Chem. 2017 Jul 26;5:54. doi: 10.3389/fchem.2017.00054 (PMC5526924; doi:10.3389/fchem.2017.00054)
Supplement: Supplementary file 1 [file DataSheet1.DOCX]

**Analysis of urine composition in type Ⅱ diabetic mice after intervention therapy using holothurian polypeptides**

Yanyan Li,^1,2^ Jiajie Xu,^1,3^ and Xiurong Su^1*^

(^1^School of Marine Science, Ningbo University, Zhejiang, China, ^2^Department of Food Science, Cornell University, Ithaca, NY 14853, USA, ^3^College of Engineering, China Agricultural University, Beijing, China.)

Correspondence to: [suxiurong@nbu.edu.cn](mailto:suxiurong@nbu.edu.cn)

**Supplementary Information:**

Tables S1-S4

Figures S1

**Table S1** The component of the holothurian (n=6, %, dry weight)

| Component | Crude protein | Crude fat | Ash | Salinity | Crude carbohydrate |
| --- | --- | --- | --- | --- | --- |
| *Acaudina molpadioides* | 54.80 | 0.42 | 22.05 | 7.79 | 1.12 |
| *Apostichopus japonicus* | 52.08 | 3.27 | 28.93 | 15.41 | 1.05 |

**Table S2** Volatile compounds in the urine of Db/Db mice by SPME and GC-MS. C: control group; M: model group; Me: positive drug group;A: A. molpadioides's polypeptide group; S: A. japonicus's polypeptide group. --: No detected

| Compounds | C | M | ME | A | S |
| --- | --- | --- | --- | --- | --- |
| 2-[methyl(phenylmethyl)amino]-ethanol | 1.27 | 1.27 | 2.35 | 3.43 | 2.45 |
| α-Terpineol | 0.72 | 1.41 | 1.23 | 4.02 | 0.83 |
| 2R-Acetoxymethyl-1,3,3-trimethyl-4t-(3-methyl-2-buten-1-yl)-1t-cyclohexanol | 7.21 | 7.39 | 9.36 | 31.3 | -- |
| Cedrol | -- | -- | -- | -- | 37.9 |
| 2-Chloro-5,5-dimethyl-1-phenyl-3-hexen-1-ol | -- | 1.01 | -- | -- | -- |
| 1-Heptyn-3-ol | -- | 14.96 | 2.63 | -- | -- |
| cis-2,3,4,4a,5,6,7,8-octahydro-1,1,4a,7-tetramethyl-, 1H-Benzocyclohepten-7-ol | -- | -- | -- | 4.78 | -- |
| 3,7-dimethyl-1,6-Octadien-3-ol | 1.61 | -- | -- | -- | -- |
| (1R-exo)-7-ethyl-5-methyl-6,8-Dioxabicyclo[3.2.1]octane | 1.36 | 0.90 | 0.74 | 1.84 | 1.00 |
| 7-Exo-ethyl-5-methyl-6,8-dioxabicyclo[3.2.1]oct-3-ene | 19.41 | 16.92 | 6.96 | 10.5 | 6.11 |
| 3-ethyl-4,4-dimethyl-2-Pentene | 8.29 | 2.57 | 2.16 | -- | 3.99 |
| 1-iodo-Tridecane | 1.45 | -- | -- | 3.27 | 1.46 |
| Hexadecane | -- | -- | 0.99 | -- | 1.31 |
| [S-(R*,S*)]-5-(1,5-dimethyl-4-hexenyl)-2-methyl  -1,3-Cyclohexadiene | -- | -- | 0.46 | -- | 0.65 |
| 2-iodo-Pentane | -- | -- | 1.75 | 2.97 | -- |
| 1-iodo-tetradecane | 0.60 | 0.96 | -- | -- | -- |
| 3,3-dimethyl-pentane | -- | -- | -- | 1.4 | -- |
| 1-iodo-Dodecane | -- | 1.47 | -- | -- | -- |
| Dimethyldiazene | 1.55 | -- | -- | -- | -- |
| 1-Hexyl-2-nitrocyclohexane | -- | -- | 1.66 | -- | -- |
| 1-Iodo-2,3-epoxypropane | -- | -- | 2.27 | -- | -- |
| 1-Formyl-2,2,6-trimethyl-3-cis-(3-methylbut-2-enyl)-5-cyclohexene | -- | 0.69 | -- | -- | -- |
| [S-(Z,E)]-1,5-Cyclodecadiene-1,5-dimethyl-  8-(1-methylethenyl) | -- | -- | -- | -- | 1.81 |
| 4-Amino-4,5(1H)-dihydro-1,2,4-triazole-5-one | 1.47 | 1.38 | 1.55 | 1.69 | 1.06 |
| 6-ethyl-7-hydroxy-4-Octen-3-one | 11.8 | 2.85 | 1.35 | 4.36 | 0.57 |
| 2-Pentanone | 3.35 | -- | 0.60 | 1.68 | 1.25 |
| 5-Hexen-3-one | 2.35 | 1.83 | 1.33 | -- | 7.33 |
| Acetone | -- | 6.0 | -- | -- | -- |
| 2-hydroxy-3,4-dimethyl-2-Cyclopenten-1-one | 1.75 | 0.59 | -- | -- | -- |
| 2-Heptanone | 2.48 | -- | -- | -- | -- |
| 6-methyl-3-Heptanone | 0.59 | -- | -- | -- | -- |
| 3-Hepten-2-one | 0.60 | -- | -- | -- | -- |
| 6-methyl-5-Hepten-2-one | 2.19 | -- | -- | -- | -- |
| 2-amino-2,4,6-Cycloheptatrien-1-one | 1.39 | -- | -- | -- | -- |
| 1-phenyl-1,2-Propanedione, | 3.8 | -- | -- | -- | -- |
| 2-Undecanone | -- | 11.32 | -- | -- | -- |
| 3-Penten-2-one | -- | 1.39 | -- | -- | -- |
| 4-Tetradecanone | -- | -- | -- | -- | 6.08 |
| 2-nitro-Benzaldehyde | -- | -- | 1.46 | -- | -- |
| 2-Trifluoromethylbenzoic acid, 6-ethyl-3-octyl ester | 2.30 | 1.75 | -- | 1.55 | 1.91 |
| Propanoic acid, 2-methyl-, 1-(1,1-dimethylethyl)-2-methyl-1,3-propanediyl ester | 1.58 | 3.57 | -- | 4.98 | -- |
| D-Alanine, N-(2,5-difluorobenzoyl)-, decyl ester | -- | -- | 23.90 | 10.91 | 22.8 |
| 2,6-Difluoro-3-methylbenzoic acid- eicosyl ester | -- | 0.75 | 0.76 | -- | -- |
| Ethyl mandelate | -- | 10.5 | -- | 2.13 | -- |
| Diethyl 1-amino-1-phenylmethanephosphonate | -- | 0.80 | -- | -- | -- |
| Fumaric acid propyl tetrahydrofurfuryl ester | -- | -- | 23.4 | -- | -- |
| Cyclohexanemethyl propanoate | -- | -- | 7.75 | -- | -- |
| [3R-(3α,3aβ,7β,8aα)]-2,3,4,7,8,8a-hexahydro-3,6,8,8-  tetramethyl-1H-3a,7-Methanoazulene | -- | -- | 0.61 | 1.35 | 0.77 |
| 5-propyl-1,3-Benzodioxole | -- | 0.50 | 1.15 | -- | -- |
| Benzoyl iso-thiocyanate | 6.55 | -- | -- | 3.27 | -- |
| 3-methyl-Hexanenitrile | 0.91 | -- | 0.85 | -- | -- |
| 2,4-bis(1,1-dimethylethyl)-Phenol | 1.59 | -- | -- | 0.47 | -- |
| Acetophenone | 0.99 | -- | -- | -- | -- |
| 2-Amino-5-propyl-1,3,4-thiadiazole | 10.8 | -- | -- | -- | -- |
| Hexanenitrile | -- | 6.32 | -- | -- | -- |
| 4-ethyl-Phenol | -- | -- | -- | 4.09 | -- |
| (1α,3aα,7α,8aβ)-octahydro-1,9,9-trimethyl-4-methylene-1H-3a-7-Methanoazulene | -- | 0.83 | -- | -- | -- |
| Menadione | -- | -- | 0.90 | -- | -- |
| 1-[(3,4-dimethylbenzoyl)oxy]-2,5-Pyrrolidinedione | -- | -- | 1.80 | -- | -- |

**Table S3** Ingredient of Feed

| Name | Content (%) |
| --- | --- |
| Corn Powder | 40-43 |
| Bran | 26 |
| Soya-bean Cake | 29 |
| Common Salt | 1 |
| Bone Powder | 1 |
| Lysine | 1 |
| Vitamin | 1 |

**Table S4** Protein information for the unknown electrophoresis dot with the MASCOT analysis from different groups. C: control group; M: model group; Me: positive drug group; A: *A. molpadioides's* polypeptide group; S: *A. japonicus's* polypeptide group

| Spot No. | Mascot score | Theorectical MW(kDa)/PI | Protein name | Species |
| --- | --- | --- | --- | --- |
| A01 | 348 | 17720/4.85 | Major urinary proteins 11 and 8 (Fragment) OS=Mus musculus GN=Mup8 PE=1 SV=1 | [Mus musculus](http://www.ncbi.nlm.nih.gov/Taxonomy/Browser/wwwtax.cgi?lvl=0&id=10090) |
| A02 | 79 | 17720/4.85 | Major urinary proteins 11 and 8 (Fragment) OS=Mus musculus GN=Mup8 PE=1 SV=1 | [Mus musculus](http://www.ncbi.nlm.nih.gov/Taxonomy/Browser/wwwtax.cgi?lvl=0&id=10090) |
| A05 | 122 | 70700/5.75 | Serum albumin OS=Mus musculus GN=Alb PE=1 SV=3 | [Mus musculus](http://www.ncbi.nlm.nih.gov/Taxonomy/Browser/wwwtax.cgi?lvl=0&id=10090) |
| A09 | 131 | 26871/4.40 | Anionic trypsin-2 OS=Mus musculus GN=Prss2 PE=2 SV=1 | [Mus musculus](http://www.ncbi.nlm.nih.gov/Taxonomy/Browser/wwwtax.cgi?lvl=0&id=10090) |
| A11 | 220 | 70700/5.75 | Serum albumin OS=Mus musculus GN=Alb PE=1 SV=3 | [Mus musculus](http://www.ncbi.nlm.nih.gov/Taxonomy/Browser/wwwtax.cgi?lvl=0&id=10090) |
| A14 | 221 | 70700/5.75 | Serum albumin OS=Mus musculus GN=Alb PE=1 SV=3 | [Mus musculus](http://www.ncbi.nlm.nih.gov/Taxonomy/Browser/wwwtax.cgi?lvl=0&id=10090) |
| A16 | 131 | 70700/5.75 | Serum albumin OS=Mus musculus GN=Alb PE=1 SV=3 | [Mus musculus](http://www.ncbi.nlm.nih.gov/Taxonomy/Browser/wwwtax.cgi?lvl=0&id=10090) |
| A19 | 485 | 70700/5.75 | Serum albumin OS=Mus musculus GN=Alb PE=1 SV=3 | [Mus musculus](http://www.ncbi.nlm.nih.gov/Taxonomy/Browser/wwwtax.cgi?lvl=0&id=10090) |
| A20 | 349 | 70700/5.75 | Serum albumin OS=Mus musculus GN=Alb PE=1 SV=3 | [Mus musculus](http://www.ncbi.nlm.nih.gov/Taxonomy/Browser/wwwtax.cgi?lvl=0&id=10090) |
| C01 | 220 | 21736/4.80 | Major urinary protein 3 OS=Mus musculus GN=Mup3 PE=1 SV=1 | [Mus musculus](http://www.ncbi.nlm.nih.gov/Taxonomy/Browser/wwwtax.cgi?lvl=0&id=10090) |
| C03 | 256 | 21736/4.80 | Major urinary protein 3 OS=Mus musculus GN=Mup3 PE=1 SV=1 | [Mus musculus](http://www.ncbi.nlm.nih.gov/Taxonomy/Browser/wwwtax.cgi?lvl=0&id=10090) |
| C05 | 279 | 17720/4.85 | Major urinary proteins 11 and 8 (Fragment) OS=Mus musculus GN=Mup8 PE=1 SV=1 | [Mus musculus](http://www.ncbi.nlm.nih.gov/Taxonomy/Browser/wwwtax.cgi?lvl=0&id=10090) |
| C10 | 115 | 70700/5.75 | Serum albumin OS=Mus musculus GN=Alb PE=1 SV=3 | [Mus musculus](http://www.ncbi.nlm.nih.gov/Taxonomy/Browser/wwwtax.cgi?lvl=0&id=10090) |
| C11 | 384 | 70700/5.75 | Serum albumin OS=Mus musculus GN=Alb PE=1 SV=3 | [Mus musculus](http://www.ncbi.nlm.nih.gov/Taxonomy/Browser/wwwtax.cgi?lvl=0&id=10090) |
| C17 | 191 | 70700/5.75 | Serum albumin OS=Mus musculus GN=Alb PE=1 SV=3 | [Mus musculus](http://www.ncbi.nlm.nih.gov/Taxonomy/Browser/wwwtax.cgi?lvl=0&id=10090) |
| ME02 | 86 | 70700/5.75 | Serum albumin OS=Mus musculus GN=Alb PE=1 SV=3 | [Mus musculus](http://www.ncbi.nlm.nih.gov/Taxonomy/Browser/wwwtax.cgi?lvl=0&id=10090) |
| ME04 | 82 | 70700/5.75 | Serum albumin OS=Mus musculus GN=Alb PE=1 SV=3 | [Mus musculus](http://www.ncbi.nlm.nih.gov/Taxonomy/Browser/wwwtax.cgi?lvl=0&id=10090) |
| ME06 | 351 | 70700/5.75 | Serum albumin OS=Mus musculus GN=Alb PE=1 SV=3 | [Mus musculus](http://www.ncbi.nlm.nih.gov/Taxonomy/Browser/wwwtax.cgi?lvl=0&id=10090) |
| ME08 | 265 | 70700/5.75 | Serum albumin OS=Mus musculus GN=Alb PE=1 SV=3 | [Mus musculus](http://www.ncbi.nlm.nih.gov/Taxonomy/Browser/wwwtax.cgi?lvl=0&id=10090) |
| ME11 | 256 | 70700/5.75 | Serum albumin OS=Mus musculus GN=Alb PE=1 SV=3 | [Mus musculus](http://www.ncbi.nlm.nih.gov/Taxonomy/Browser/wwwtax.cgi?lvl=0&id=10090) |
| ME14 | 315 | 70700/5.75 | Serum albumin OS=Mus musculus GN=Alb PE=1 SV=3 | [Mus musculus](http://www.ncbi.nlm.nih.gov/Taxonomy/Browser/wwwtax.cgi?lvl=0&id=10090) |
| ME15 | 79 | 20935/5.04 | Major urinary protein 2 OS=Mus musculus GN=Mup2 PE=1 SV=1 | [Mus musculus](http://www.ncbi.nlm.nih.gov/Taxonomy/Browser/wwwtax.cgi?lvl=0&id=10090) |
| ME16 | 423 | 28167/5.52 | Ela3 protein, partial [Mus musculus] | [Mus musculus](http://www.ncbi.nlm.nih.gov/Taxonomy/Browser/wwwtax.cgi?lvl=0&id=10090) |
| ME17 | 61 | 70700/5.75 | Serum albumin OS=Mus musculus GN=Alb PE=1 SV=3 | [Mus musculus](http://www.ncbi.nlm.nih.gov/Taxonomy/Browser/wwwtax.cgi?lvl=0&id=10090) |
| ME19 | 211 | 39916/5.96 | Protein AMBP OS=Mus musculus GN=Ambp PE=1 SV=2 | [Mus musculus](http://www.ncbi.nlm.nih.gov/Taxonomy/Browser/wwwtax.cgi?lvl=0&id=10090) |
| ME20 | 433 | 70700/5.75 | Serum albumin OS=Mus musculus GN=Alb PE=1 SV=3 | [Mus musculus](http://www.ncbi.nlm.nih.gov/Taxonomy/Browser/wwwtax.cgi?lvl=0&id=10090) |
| ME22 | 416 | 70700/5.75 | Serum albumin OS=Mus musculus GN=Alb PE=1 SV=3 | [Mus musculus](http://www.ncbi.nlm.nih.gov/Taxonomy/Browser/wwwtax.cgi?lvl=0&id=10090) |
| ME23 | 537 | 70700/5.75 | Serum albumin OS=Mus musculus GN=Alb PE=1 SV=3 | [Mus musculus](http://www.ncbi.nlm.nih.gov/Taxonomy/Browser/wwwtax.cgi?lvl=0&id=10090) |
| ME26 | 319 | 70700/5.75 | Serum albumin OS=Mus musculus GN=Alb PE=1 SV=3 | [Mus musculus](http://www.ncbi.nlm.nih.gov/Taxonomy/Browser/wwwtax.cgi?lvl=0&id=10090) |
| ME27 | 183 | 20935/5.04 | Major urinary protein 2 OS=Mus musculus GN=Mup2 PE=1 SV=1 | [Mus musculus](http://www.ncbi.nlm.nih.gov/Taxonomy/Browser/wwwtax.cgi?lvl=0&id=10090) |
| ME32 | 366 | 70700/5.75 | Serum albumin OS=Mus musculus GN=Alb PE=1 SV=3 | [Mus musculus](http://www.ncbi.nlm.nih.gov/Taxonomy/Browser/wwwtax.cgi?lvl=0&id=10090) |
| ME34 | 319 | 70700/5.75 | Serum albumin OS=Mus musculus GN=Alb PE=1 SV=3 | [Mus musculus](http://www.ncbi.nlm.nih.gov/Taxonomy/Browser/wwwtax.cgi?lvl=0&id=10090) |
| ME35 | 395 | 70700/5.75 | Serum albumin OS=Mus musculus GN=Alb PE=1 SV=3 | [Mus musculus](http://www.ncbi.nlm.nih.gov/Taxonomy/Browser/wwwtax.cgi?lvl=0&id=10090) |
| ME37 | 167 | 70700/5.75 | Serum albumin OS=Mus musculus GN=Alb PE=1 SV=3 | [Mus musculus](http://www.ncbi.nlm.nih.gov/Taxonomy/Browser/wwwtax.cgi?lvl=0&id=10090) |
| ME38 | 121 | 70700/5.75 | Serum albumin OS=Mus musculus GN=Alb PE=1 SV=3 | [Mus musculus](http://www.ncbi.nlm.nih.gov/Taxonomy/Browser/wwwtax.cgi?lvl=0&id=10090) |
| ME39 | 349 | 70700/5.75 | Serum albumin OS=Mus musculus GN=Alb PE=1 SV=3 | [Mus musculus](http://www.ncbi.nlm.nih.gov/Taxonomy/Browser/wwwtax.cgi?lvl=0&id=10090) |
| ME40 | 80 | 70700/5.75 | Serum albumin OS=Mus musculus GN=Alb PE=1 SV=3 | [Mus musculus](http://www.ncbi.nlm.nih.gov/Taxonomy/Browser/wwwtax.cgi?lvl=0&id=10090) |
| S01 | 113 | 20935/ 5.04 | Major urinary protein 2 OS=Mus musculus GN=Mup2 PE=1 SV=1 | [Mus musculus](http://www.ncbi.nlm.nih.gov/Taxonomy/Browser/wwwtax.cgi?lvl=0&id=10090) |
| S02 | 268 | 70700/5.75 | Serum albumin OS=Mus musculus GN=Alb PE=1 SV=3 | [Mus musculus](http://www.ncbi.nlm.nih.gov/Taxonomy/Browser/wwwtax.cgi?lvl=0&id=10090) |
| S03 | 335 | 70700/5.75 | Serum albumin OS=Mus musculus GN=Alb PE=1 SV=3 | [Mus musculus](http://www.ncbi.nlm.nih.gov/Taxonomy/Browser/wwwtax.cgi?lvl=0&id=10090) |
| S07 | 66 | 70700/5.75 | Serum albumin OS=Mus musculus GN=Alb PE=1 SV=3 | [Mus musculus](http://www.ncbi.nlm.nih.gov/Taxonomy/Browser/wwwtax.cgi?lvl=0&id=10090) |
| S09 | 280 | 20935/5.04 | Major urinary protein 2 OS=Mus musculus GN=Mup2 PE=1 SV=1 | [Mus musculus](http://www.ncbi.nlm.nih.gov/Taxonomy/Browser/wwwtax.cgi?lvl=0&id=10090) |
| S10 | 230 | 70700/5.75 | Serum albumin OS=Mus musculus GN=Alb PE=1 SV=3 | [Mus musculus](http://www.ncbi.nlm.nih.gov/Taxonomy/Browser/wwwtax.cgi?lvl=0&id=10090) |
| S16 | 92 | 17720/4.85 | Major urinary proteins 11 and 8 (Fragment) OS=Mus musculus GN=Mup8 PE=1 SV=1 | [Mus musculus](http://www.ncbi.nlm.nih.gov/Taxonomy/Browser/wwwtax.cgi?lvl=0&id=10090) |
| S17 | 286 | 70700/5.75 | Serum albumin OS=Mus musculus GN=Alb PE=1 SV=3 | [Mus musculus](http://www.ncbi.nlm.nih.gov/Taxonomy/Browser/wwwtax.cgi?lvl=0&id=10090) |
| S19 | 141 | 45915/7.14 | Napsin-A OS=Mus musculus GN=Napsa PE=1 SV=1 | [Mus musculus](http://www.ncbi.nlm.nih.gov/Taxonomy/Browser/wwwtax.cgi?lvl=0&id=10090) |


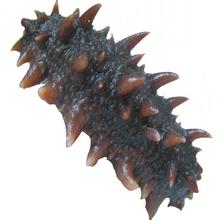

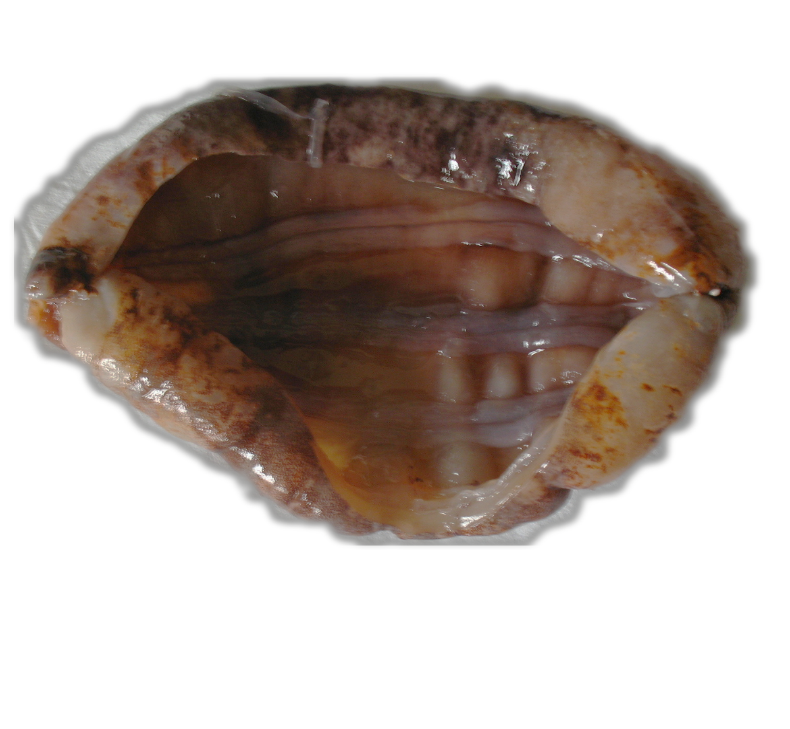


**Figure S1** Photographs of the body wall of *A. molpadioides* (left) and *A. japonicus* (right).
